# Impact of self management on metabolic control indicators of diabetes patients

**DOI:** 10.1186/2251-6581-11-6

**Published:** 2012-08-24

**Authors:** Marzieh Moattari, Akram Ghobadi, Parvin Beigi, Gholamreza Pishdad

**Affiliations:** 1 Fatemeh (PBUH) School of Nursing & Midwifery, Shiraz University of Medical Sciences, Zand blvd, Po. Box 71345-1359, 71936-13119, Shiraz, Iran; 2 School of Nursing & Midwifery, Kermanshah University of Medical Sciences, Isar Sq, Dolatabad, Kermanshah, Iran; 3 Department of Internal Medicine, Medical School, Shiraz University of Medical Sciences, Shiraz, Iran

**Keywords:** Self management, Education, Hemoglobin A_1_c, Patients with diabetes, Iran

## Abstract

**Introduction:**

Patients with diabetes play an important role in management of their illness. They should be involved in the intervention program to be able to improve their quality of life. This study investigated the effect of a self-management program based on 5A (assess, advise, agree, assist, arrange) model on metabolic control indicators of diabetic patients.

**Material and method:**

In this randomized, controlled trial, 70 diabetic insulin dependent patients who referred to Nader Kazemi clinic in Shiraz participated. They were randomly assigned to two groups of experiment and control. Then a self-management program based on 5A model was performed for the experiment group during 3-mounths and the indicators of metabolic control including hemoglobin A_1_c, cholesterol, triglyceride and also body mass index were measured pre- and post- intervention in both groups. Data were analyzed using SPSS ver 11.5.

**Results:**

Data analysis revealed a significant reduction in mean fasting blood sugar (69 units) and HbA_1_c (1.16 units) in the experimental group . Pre- to post-changes in hemoglobin A_1_c, fasting blood sugar, and high density lipoprotein were significant between the two groups of study. However, there was no significant difference in cholesterol, triglyceride, low-density lipoprotein, and body mass index between the two groups.

**Discussion:**

Regarding the results, it can be concluded that a 3-month self-management program based on 5A model is effective in reducing the fasting blood sugar, hemoglobin A_1_c. Application of self management program based on 5A model in diabetic clinics is recommended.

## Introduction

The prevalence of diabetes has a growing trend in the world as well as in Iran. In addition, it is the cause of the mortality [1] and morbidity [2]. Although the control of diabetes and its complications is costly [3], it has been shown that glycemic control decreases the complications of diabetes [4] so that it could be identified as the main predictor of chronic complications of diabetes [5]. Therefore, maintaining hemoglobin HbA_1_c (HbA_1_c) within the normal range should be considered as the goal of treating diabetes [6]. It has been shown that a 1% reduction in HbA_1c_ for 10 years results in a 21-percent reduction in deaths related to diabetes and its complications [1].

Furthermore, association of diabetes with dislipidemia [5,7] and the fact that diagnosis and treatment of dislipidemia leads to reduction of coronary vascular disease (CVD) and mortality [5] make health care providers consider management of dislipidemia in diabetes patients’ management programs.

Effective care, treatment and control of diabetic patients depend on the participation of the patients and their families in self management programs [2]. However, traditional teaching methods which lack the above mentioned feature are predominant in diabetic patient’s education programs. Such methods of education increase the patient’ knowledge but they have little clinical value. It has been found that the use of multi-method educational interventions will result in a better outcome in diabetic patients compared with the interventions in which just one method (education or behavior modification) is used [8].

While different models of self management/education create appropriate strategies for changing behavior, researchers have paid little attention to application of self management models in diabetic patients’ education. The need for investigating the impact of the self management diabetes education delivery format on diabetes health-related outcomes has been highlighted by Tang, et al. (2006) [9].

Five A model, known as behavior change counseling model is an evidence-based approach for behavior change and health promotion [10]. This model includes assessment of behavior, beliefs and motivation of the patient (Assess); provide information about personal health risks and benefits of change (Advise); mutual contribution of patients and health care provider on setting realistic goals based on the patient’s interest and confidence in their ability to change the behavior (Agree); help anticipate barriers and develop practical applications based on the identified patient’s strategies, problem-solving techniques and social/environmental support (Assist) and specify plan for follow-up (e.g., Visits, Phone calls) and providing support in the course of follow up (Arrange) [11-13]. This model was used for the first time in Iran in a research conducted on post-coronary artery bypass graft (CABG) patients and its effectiveness was confirmed [14]. Therefore, considering the weaknesses of traditional teaching strategies in involving patients to actively participate in their treatment process and the necessity to help and support them during behavior change process, we used the 5A model as a conceptual framework for self management to find out its effect on metabolic control of insulin dependent diabetic patients.

## Methods

This is a randomized trial study aiming to examine the effect of 5A self management model on metabolic control indicators including fasting blood sugar, HbA_1c_, cholesterol, triglyceride, lipoprotein (HDL and LDL) and body mass index. After obtaining the approval of the ethics committee and diabetic association and also the patient’s informed consent, the convenience and a purposeful sampling method helped randomly divide the participants into two experimental and control groups. All the patients met the inclusion criteria as insulin dependent diabetes diagnosis, aged 18-40 years, guidance school education, ability to comprehend educational material, and ability to attend the educational program of the study. Exclusion criteria were taking part in previous education program and having psychiatric disorder. a convenient sample of 82 patients were involved in the study They were randomly assigned in experimental and control groups. Blood samples were drawn after at least 12.00 hours of overnight fasting. Fasting blood sugar, HbA_1_c, lipid profile including cholesterol, triglyceride, lipoprotein (HDL and LDL) and body mass index: weight (kg)/height (m)^2^ were measured as follows.

## Glucose assay

The glucose assay was performed by colorimetric enzyme method (Trinder 1969). In this method, glucose with effect of Glucose oxidase can be enzymatically oxidized to gluconic acid and hydrogen peroxide in the presence of peroxidase. The hydrogen peroxide reacts with 4-amino antipyrine (4- AAP) and N-ethyl-N-sulfopropyl-m-toluidine (TOPS) to form violet-colored quinoneimine, which has an absorbance peak at 520 nm [15].

## HbA_1_c

Total Hb and HbA_1_c concentrations are determined after hemolysis of the anticoagulated whole blood specimen. Total Hb is measured colormetrically. HbA_1_c is determined immunoturbicimetrically. The ratio of both concentration yields the final percent HbA_1_c result [HbA_1_c (%)].

## Plasma total cholesterol, triglycerides, HDL and LDL

Cholesterol is determined by enzymatic, colorimetric method (CHOD/PAP) with cholesterol esterase, cholesterol oxidase, and 4-aminoantipyrine. Triglyceride levels are determined by enzymatic, colorimetric method (GPO/PAP) with glycerol phosphate oxidase and 4-aminophenazone. HDL was measured by immunoinhibitory method.

All the required measures for correct weighing of the patients were considered.

## Intervention of the study

A self management model called 5A model was applied for the experimental group during a 3 month period in the following stages based on each individual’s needs. In the first stage of the intervention (Assess), the patients were interviewed and their behavior on insulin injection, use of hypoglycemic drug, blood sugar self monitoring, exercise/physical activity and foot care was assessed. Also, the test results were considered as a reflection of patient status and as motivating factors to patient’s behavioral changes. Also, their beliefs and motivation regarding the life style change were explored.

In the second stage (Advise), all the abnormal or unexpected findings obtained from the assessment stage were reviewed with the patients. Also, all the health related risks were identified and shared with the patients. Benefits of behavioral change and its relation to their health were emphasized. Acute and chronic complication of diabetes, risk factors and preventive measures were explained. The importance of behavior change in prevention or delaying the onset of complications was highlighted.

In the 3^rd^ stage: (Agree), a written agreement between the patient and healthcare provider about the patient’s necessary performance was developed. Appropriate behavioral objectives and action plans for each objective were included in this agreement based on patients’ condition, interest and priority in relation to the findings from his/her assessment. Different options/choices were identified based on the input received from the patients, his/her significant other and the caregiver. Patients were asked to rate their efficacy to commit each of the agreed upon action plan. Also, they were asked to report their responses (self report) to the behavioral change plan on daily basis [16] for the 12 weeks of intervention

In the fourth stage (Assist), the patients’ self efficacy for each action plan was evaluated. Also, barriers to commit the action plan and the ways to overcome barriers were explored. Educational pamphlets containing necessary information and hints about foot care, insulin injection, and blood glucose self monitoring, nutrition, and exercise were provided to them. These pamphlets have been designed by Iranian diabetic association and are commonly available for these patients. Also, a nutrition counseling session based on the associated findings was held for each individual patient. Furthermore, they were given a tape on stress management and muscle relaxation. To overcome financial barriers for self monitoring of blood glucose, the patients received strips for measurement of blood glucose. Although the intervention of the study was mostly individual, group sessions of three to four individuals were held for patients if the identified problems were common among them. These group sessions were beneficial to make the patients aware of the social supports available in the community. During follow-up visits, progress, experience, concerns were reviewed and the unmet objectives were renegotiated, and action plans were revised. Cultural background of the patients was respected during these negotiations.

In the fifth stage (Arrange) which continued during the 12 weeks of the intervention, all the patients were followed up by phone calls and/or in person if required. Their success or failure to commit the action plans and meeting the objectives were assessed and necessary approved changes were made in the action plan. Arrangement for the patient to be in contact with specific community resources that could support him/her and special arrangements for referral were made. These sessions were not longer than half an hour.

Institutional Review Board (IRB) approval for the study was obtained from the Ethics Committee of Shiraz University of Medical Sciences (ECSUMS). Written consent was obtained from each patient. The purpose of the study, voluntary participation, confidentiality and freedom to discontinue at any time without being left untreated was reviewed. The study was carried out in Nader Kazemi Diabetic clinic in Shiraz located in Fars province, southern Iran. This is the main diabetic clinic in Shiraz affiliated with Shiraz University of Medical Sciences. This centre offers services (treatment and follow up) to at least 50 patients on a daily basis.

Totally 70 patients completed the study. The collected data were analyzed in SPSS software ver 11.5, using Chi-square tests, paired and independent *t*-test for assessing fasting blood sugar, hemoglobin A_1C_, cholesterol, triglyceride, lipoprotein (HDL and LDL) and body mass index before and after the intervention in each and between groups.

## Results

The participants (N = 70) were aged between 18-40 (mean = 24.13) years and their duration of diabetes was 1-23 years (mean = 9). Table 1 presents other demographic characteristics of the participants. Most of the participants were female, married, and jobless, with negative family history of diabetes, high school educational level and without co-morbidity. There was no significant difference in the demographic variables between the two groups with respect to age, marital status, and educational level, type of diabetes, job, positive family history and co-morbidity.

**Table 1:** Demographic characteristics of patients

**(N = 70)**	
**Sex** (No&%)	
Male	(50)70.42
Female	(20)29.58
**Marital status** (No&%)	
Married	(53)75.7
other	(17)24.3
**Occupation** (No&%)	
Housekeeper	(13)18.57
Student	
University	(15)21.42
high school	(6)8.57
Unemployed	(19)27.14
Self employed	(13)18.57
Employee	(4)5.71
**Type of diabetes** (No&%)	
Type1	(68)97.14
Type2	(2)2.86
**Education level** (No&%)	
Guidance school	(17)24.29
High school	(32)45.71
University	(21)30
**Family history of diabetes** (No&%)	
Negative history	(29)41.43
Positive history	(41)58.57
Sibling	(13)18.57
parents	(9)12.86
others	(19)27.14
**Co morbidity** (No&%)	
None	(55)78.57
Thyroid	(10)14.29
Thalassemia	(5)7.14

Assessing the results before interventions using independent *t*-test showed that the two groups were similar in fasting blood sugar, hemoglobin A_1c_, cholesterol, triglyceride, lipoprotein (HDL and LDL) and body mass index and there was no statistically significant difference between the two groups before the intervention.

The results regarding the two groups’ pre- to post-changes in fasting blood sugar, hemoglobin A_1c_, cholesterol, triglyceride, lipoprotein (HDL and LDL) and body mass index indicated a significant improvement in the experimental group compared to the control group in regard to fasting blood sugar, hemoglobin A_1c_, and HDL. No specific difference was seen between the two groups in cholesterol, triglyceride, lipoprotein LDL and body mass index (Table 2).

**Table 2:** **Mean values of FBS, lipid profile, HbA**_**1c**_**and BMI before and after the intervention in both groups (Data are mean ± SD)**

	**Experimental Group (n = 35)**		**Control Group (n = 35)**	
**Variables**	**Before treatment**	**After treatment**	**Before treatment**	**After treatment**
FBS (mg/dl) *	196 ± 100	127 ± 67	155 ± 83	196 ± 120
TG (mg/dl) **	120 ± 54.55	108 ± 65.27	113 ± 43	138 ± 91
TC (mg/dl) **	172 ± 25.78	172 ± 27.83	165 ± 32	178. ± 52
LDL (mg/dl) **	107 ± 21.75	109 ± 24.91	102 ± 26	112 ± 35
HDL (mg/dl) *	42 ± 7.24	43 ± 9.45	42 ± 5.44	38 ± 8.04
HbA_1c_ (%)*	8.24 ± 1.44	7.08 ± 1.26	8.3 ± 1.52	8.18 ± 2.05
BMI **	20.99 ±2.65	21.09 ± 2.54	22.55 ± 4	22.87 ± 4

P*<0.05 p**>0.05 between two groups comparisons.

*FBS*, fasting blood sugar; *TG*, triglycerides; *TC*, total cholesterol; *LDL*, *HDL*, low and high density, Lipoprotein ; *HbA*_*1c*_, glycated Hemoglobin; *BMI*, body mass index.

## Discussion

The results of this study support the effectiveness of using the 5A model of self management in the improvement of two important glycemic control indicators including fasting blood sugar and HbA_1c_. Considering the results in the control group, we can specify that the regular interventions existing in our diabetic clinic do not improve the glycemic control indicators of the patients. Therefore, the difference found between the two groups can be attributed to the self management model used in this study. It seems that the interactive, contributive counseling approach of the intervention is responsible for this change. As previously stated, 5A model includes a comprehensive behavior, belief and motivation assessment, individual counseling, provision of educational as well as social support resource, mutual contribution of both patient and the health care provider in reaching the agreement on specific behavioral objectives and appropriate action plans, helping the patients to take responsibility of managing the behaviors through self report notes and continuous communication with the patients during follow up period and periodic evaluation of commitments of patients to agreed upon action plans. All these features along with helping and supporting them through decision making process make the patients feel self confident and play an active role in disease management. Successful application of such educational model requires both patients and health care providers to develop a positive attitude toward it. However, it is stated that although self-management models improve health outcomes and reduce health care costs, the principles have rarely been applied in low vision services [17].

Improvement of glycemic control found in this study seems to be due to behavioral change of patients as a result of the intervention applied in the study. Application of the 5A model as a conceptual framework for the educational intervention used in this study was also previously considered in another quasi experimental study which was aimed at determining its effect on life style, body mass index and cholesterol in post coronary bypass graft patients. . Researchers concluded that all aspects of life style including health responsibility, nutrition, exercise, interpersonal communication/support and stress management but spirituality (self actualization) were improved. Also cholesterol, HDL and LDL and body mass index improved as well [14]. The findings of another study demonstrated the efficacy of the educational program in improving diabetes self-care management skills in addition to HbA_1c_[18].

The effectiveness of the model applied in the study was approved in the improvement of HDL. However, the results do not support its effectiveness in improving triglyceride, LDL and body mass index. This insignificant finding could be due to the difficulties in complying diet and difficulties and complexities in reducing weight, thereby affecting body mass index in a 3 month period. However, it should be noted that the mean body mass index of both groups of the study is within normal limits. This finding is related to the nature of type1 diabetes, from which most of the patients of this study are suffering.

Scarcity of the research applying 5A model makes it difficult to compare the results of this study with similar studies. However, other kinds of self management have been used in other studies. In a study, a culturally based self management was used for type 2 diabetes. The intervention of the study consisted of weekly patient education for three months and fortnight support group for six months. Researchers reported a significant change in fasting blood sugar, HbA_1c_ and knowledge of the patients after one year [19]. Comparing the result of the present study with this result highlights the value of the 5A model as our results were found just after 3 months of the intervention. However, the difference of the population of these two studies should not be disregarded. Type 1 diabetes patients are younger than those having type 2 and therefore they might be more motivated to maintain their healthy conditions. In another research, Kaplan (1997) conducted a clinical trial study and confirmed the effectiveness of an educational program based on American Diabetic Association in reducing HbA_1c_[20]. There is some evidence supporting the beneficial use of other types of intervention. In one study, a continuous care model was used for type 2 diabetic patients for three months. The researchers reported some improvements in both fasting blood sugar and HbA_1c_[2]. Continuous care model used in their study consists of four stages including orientation, sensitization, control and evaluation. These stages are reflecting a medical oriented approach to care in which the health care providers have authority over the patients. According to Funnell, Anderson and Robert (2004) providers have to give up the illusion that they have control of their patients’ diabetes self management decisions and outcomes [21]. Therefore, the agreement stage (goal setting) of 5A model is a unique stage through which patients involve actively in developing their self management plan. In another study, a community based intervention was used and its effectiveness was verified [22]. Also, Herenda, Tahirovic and Zildzic (2007) in their study investigated the effect of education on metabolic control of type 2 diabetic patients. They confirmed the effect of a six month education on HbA_1c_ blood pressure and cholesterol but not on triglyceride, body mass index, smoking habit and physical activity [23]. As it is discussed, the application of different models of education/self management can result in some changes in metabolic control index or lipid profile or other variables. However, it seems that the 5A model has the potential of a similar change in shorter time duration. This finding is very important when we consider time constraint and resource limitations. Furthermore, the target group of the present study was a relatively young people, indicating the generalizability of the findings to this group of patients. To generalize the findings to other groups of patients including non insulin dependent diabetes or other illnesses, repetition of the study is suggested. Also, determining the long term effect of 5A model of self management is recommended. The results related to cholesterol, LDL and triglyceride are suggestive of the necessity to modify the intervention in future studies to find out the possible effect of the model applied on change in cholesterol, LDL and triglyceride.

In general, the findings of the study revealed that the 5A model is effective in reducing the glycemic control indicators (fasting blood sugar, HbA_1c_) in insulin dependent diabetic patients.

## Competing interests

The authors declare that they have no competing interests.

## Authors’ contributions

MM devised the concept for the study, developed the study design, supervised data collection and analysis and drafted the manuscript, involved in the coordination of the study. AGh collected data, run the intervention of the study, involved in the conception of the study and performed the analyses. PB contributed in the intervention of the study. GRP contributed to the design. All authors read and approved the final manuscript.
